# Depth-Dependent High Distortion Lens Calibration

**DOI:** 10.3390/s20133695

**Published:** 2020-07-01

**Authors:** Carlos Ricolfe-Viala, Alicia Esparza

**Affiliations:** 1 Instituto de Automática e Informática Industrial, Universitat Politècnica de València, Camino de Vera s/n, 46022 Valencia, Spain; 2 Department of Systems Engineering and Automatic Control, Universitat Politècnica de València, Camino de Vera s/n, 46022 Valencia, Spain; aesparza@isa.upv.es

**Keywords:** camera calibration, high distortion, depth dependence

## Abstract

Accurate correction of high distorted images is a very complex problem. Several lens distortion models exist that are adjusted using different techniques. Usually, regardless of the chosen model, a unique distortion model is adjusted to undistort images and the camera-calibration template distance is not considered. Several authors have presented the depth dependency of lens distortion but none of them have treated it with highly distorted images. This paper presents an analysis of the distortion depth dependency in strongly distorted images. The division model that is able to represent high distortion with only one parameter is modified to represent a depth-dependent high distortion lens model. The proposed calibration method obtains more accurate results when compared to existing calibration methods.

## 1. Introduction

Lens distortion is a significant problem in the camera calibration process. This systematic error needs a precise modelling and calibration for imaging-based measurement techniques. Several different camera models have been proposed for different types of cameras. The most popular is the even-order radial distortion polynomial model that models radial distortion by means of scaling by a factor [1]. However, results using the radial distortion model are not as accurate as desired when high distortion is present. If high distortion is present, several models exist in the literature. Ricolfe-Viala in [2] compares the performance of the radial-tangential model, logarithmic, polynomial, division, and rational function distortion models with low and high distorted images under common criterion, showing that the division model and the rational function lens distortion model can represent high distortion accurately. In case of a wrong computed model, it can be refined with different methods, such as the disparity map method presented in [3]. However, better model is computed if the calibration process takes into account the model details.

**Figure 1:**
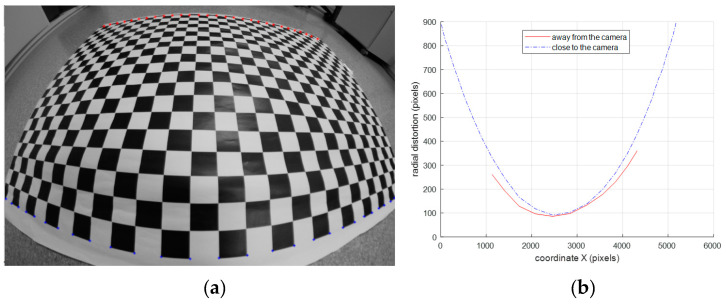
(**a**) Image of a planar checkerboard used as calibration template. The checkerboard is under perspective to notice the radial distortion variation when the camera-object distance changes. (**b**) Figure of the radial distortion profile depending on the camera-object distance. Solid red line represents radial distortion when object is away from the camera appearing at top of Image 1a. The blue dashed line represents the radial distortion when the object is close to the camera appearing at the bottom of the Image 1a.

Accurate lens distortion correction is a very complex problem since different focus implies different distortion and moreover, the distortion differs if the distance between the object and the lens varies in the depth of field range for a given focus [4]. If object location varies in the range of a sharply mapped image, deformations in the image change depending on this distance. The variations in distortion in the depth of field range are higher if fish-eye lenses are used. Figure 1 shows this strong perspective effect at diverse depth distances with respect to the camera with high distorted images. Figure 1b shows the distortion profiles of the top and down lines with respect to the center of the image and the differences are obvious. Considering a common lens distortion model for all points in the image is not very convenient if an accurate correction is needed.

The depth dependence of lens distortion model was presented by Magill in [4]. Considering the depth influence upon distortions avoid systematic measurement error. Magill demonstrated that lens distortion depends on magnification and established the radial distortion model dependent on magnification. Magill’s model computes lens distortion at any specified focus distance. However, this model is valid to points in the “in focus” depth distance only. Subsequent studies proposed magnification-dependent radial distortion models and calibration methods [5,6,7,8]. Brown [5] proposed a distortion model dependent on magnification that considers distortion outside the “in focus” distance plane when the magnitude of the distortion is small. Fryer [6] presented the decentering distortion formulation. Fraser and Shortis [6] improved the model proposed by Brown to solve the problem of small magnitudes in distortion. Alvarez [8] derived a new radial distortion model for planar scenes such as the soccer field combining Brown’s and Fraser’s models.

Based in results of Ricolfe-Viala in [3,9], the aim of this paper is to evaluate the depth dependence of the division model that is able to model high distortion accurately. First, the evaluation algorithm is described. Second, an empirical evaluation is performed. Third, a model is proposed based on the empirical evaluation results.

## 2. Materials and Methods

The image distortion model represents the mapping from the distorted image coordinates *q_d_* = (*u_d_*,*v_d_*), to the undistorted image coordinates, *q_p_* = (*u_p_,v_p_*) [10]. Distorted coordinates are observable in the images and undistorted image coordinates are not physically measurable. *r* is the distance from the point *q* = (*u,v*) to the distortion center, defined as *c_0_* = (*u*_0_, *v*_0_), and *Δu* = *u* − *u*_0_, and *Δv* = *v* − *v*_0_. *r* is computed as *r^2^ = Δu^2^ + Δv^2^*. Using the division model, the radial distortion is approximated with a polynomial as follows:

(1) $$r_{p}=\frac{r_{d}}{1+\beta_{0}\cdot r_{d}^{2}+\beta_{1}\cdot r_{d}^{4}+\ldots }$$

where *r_p_* represents the distance of the point *q_p_* = (*u_p_,v_p_*) to the distortion center and *r_d_* is the distance of the point *q_d_* = (*u_d_, v_d_*) to the distortion center. *β_i_* represent the distortion parameters. According to the degree of the polynomial in the denominator, the accuracy of the lens distortion model varies. However, the advantage of the division model over the other high lens distortion models is that it is able to express high distortion with few parameters [2]. Most of the authors have demonstrated that accurate results are computed with only one parameter for many cameras [11,12]. In the experimental results section, outcomes of one parameter model are compared with the ones of the two parameters model. The aim is to measure the improvement when the number of parameters change.

### 2.1. Division Model Calibration

**Figure 2:**
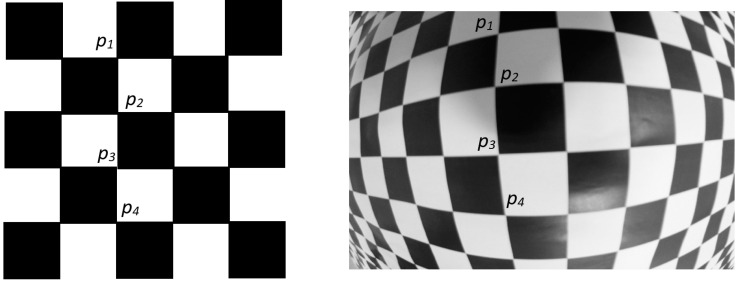
Geometric invariants for correcting points locations detected in the image. Template features remain under perspective projection also in distorted images. Cross ratio guaranties that parallel lines remain parallels under perspective projection. Straight lines are also straight under perspective projection.

The model is calibrated based on the idea of metric point correction described in [13]. The metric point correction consists of maintaining the features of the object in the image, to undistort the distorted control points. For example, control points of a checkerboard template accomplish several constraints that do not change under perspective projection. First constraint is the cross ratio (CR) between any set of four control points [13] and the second one is that the control points that belong to a straight line in the checkerboard should remain in a straight line in the image [14]. Figure 2 shows these two constraints. Using these constraints, control points in distorted images are corrected to obtain their undistorted positions. With both set of points, the distorted ones detected in the image and the undistorted ones computed using the previous constraints, the division model is computed. Cross ratio and straight line constraints for point correction are defined in the following.

#### 2.1.1. Control Points Correction

If template control points in the image with coordinates *q_1d_* = (*u_1d_*,*v_1d_*), *q_2d_* = (*u_2d_*,*v_2d_*), *q_3d_* = (*u_3d_*,*v_3d_*), *q_4d_* = (*u_4d_*,*v_4d_*) satisfy the cross-ratio invariability, the following equation arise:

(2) $$CR(q_{1d},q_{2d},q_{3d},q_{4d})=\frac{s_{13}\cdot s_{24}}{s_{14}\cdot s_{23}}=CR(p_{1},p_{2},p_{3},p_{4})$$

where *s_ij_* defines the distance between points *q_i_* and *q_j_* represented as *s_ij_*^2^ = (*u_i_* − *u_j_*)^2^ + (*v_i_* − *v_j_*)^2^; *p*_1_, *p*_2_, *p*_3_, *p*_4_ are four consecutive control points of the calibration template arranged in a line represented in Figure 2. Any set of four consecutive control points arranged in a line in the calibration template, have to satisfy the *CR*(*p*_1_, *p*_2_, *p*_3_, *p*_4_). These lines are vertical and horizontal. In consequence, one point participates in the cross ratio computing of six sets of points, three for horizontal neighbor points and three for vertical neighbor points. Using the cross ratio invariability, distorted positions of image points are corrected according to the positions of neighbor points doing a nonlinear search that minimizes the following error function:

(3) $$J_{CR}=\sum_{l=1}^{n}\sum_{k=1}^{m-3}\left\|CR(q_{k},q_{k+1,l},q_{k+2,l},q_{k+3,l})-CR(p_{1},p_{2},p_{3},p_{4})\right\|$$

Since a checkerboard template arranges control points in straight lines, *n* is the number of straight lines and *m* is the number of points in each line, *q_k,l_* is a point *k* of the straight line *l*, where *l* = 1, …, *n* and *k* = 1, …, *m*. *CR*(*p*_1_, *p*_2_, *p*_3_, *p*_4_) is computed when the calibration template is designed.

On the other side, given a control point *q_i_* = (*u_i_*, *v_i_*) in the distorted image, the straight line constraint to undistort the control point position is defined with the straight line equation.

(4) $$a_{l}\cdot u_{i}+b_{l}\cdot v_{i}+c_{l}=0$$

where *a_l_*, *b_l_*, *c_l_* represent the parameters of a line *l*. Points that fit in a line perfectly, make the following function null.

(5) $$J_{ST}=\sum_{l=1}^{n}\sum_{i=1}^{m}\left\|a_{l}\cdot u_{i}+b_{l}\cdot v_{i}+c_{l}\right\|$$

To undistort all distorted image positions an error function is minimized that includes the cross-ratio invariability and the straight line constraint.

(6) $$J_{CP}=\sum_{l=1}^{n}\left(\sum_{i=1}^{m}\left\|a_{l}\cdot u_{i}+b_{l}\cdot v_{i}+c_{l}\right\|+\sum_{k=1}^{m-3}\left\|CR(q_{k},q_{k+1,l},q_{k+2,l},q_{k+3,l})-CR(p_{1},p_{2},p_{3},p_{4})\right\|\right)$$

**Figure 3:**
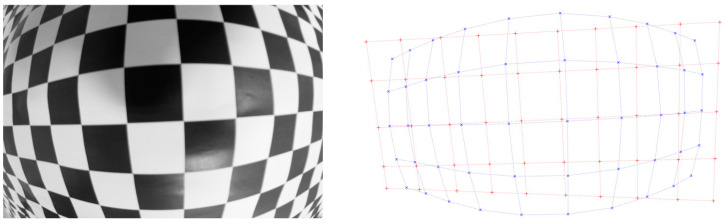
Result of control points correction. Blue dots correspond to detected distorted control points in the image *q_d,i_* and red lines correspond to the undistorted control points *q_p,i_*. Correction is done taking into account that control points of a checkerboard template accomplish two constraints that do not change under perspective projection. The first one is the cross ratio between any set of four control points and the second constraint is that control points that belong to a straight line in the checkerboard should remain in a straight line in the image.

Minimizing previous function, distorted control points *q_d,i_* are corrected to undistorted ones *q_p,i_*. Figure 3 shows the result. Blue dots correspond to detected control points in the image *q_d,i_* and red lines correspond to the undistorted control points *q_p,i_*.

#### 2.1.2. Computing the Model Parameters

The parameters of the division model described in (1) are the coefficients of the polynomial in the denominator denoted as *β_i_* and the distortion center *u*_0_, *v*_0_. Given a set of distorted points *q_d,i_* and the undistorted ones *q_p,i_*, where *i* represents the number of points in the image, *n* pairs (*r_d,i_*, *r_p,i_*) arise, which are used to compute the coefficients *β_i_*. Considering a two parameter model, two coefficients *β_i_* are arranged in a vector *β* = [*β*_0_, *β*_1_]^T^ and considering *u*_0_, *v*_0_ in the center of the image, given *n* points in the image, *β* is computed as follows:

(7) $$\left[\begin{array}{c} r_{p,1}\cdot r_{d,1}^{2}\\ \ldots \\ r_{p,n}\cdot r_{d,n}^{2} \end{array}\begin{array}{c} r_{p,1}\cdot r_{d,1}^{4}\\ \ldots \\ r_{p,n}\cdot r_{d,n}^{4} \end{array}\right]\cdot \beta =\left[\begin{array}{c} r_{d,1}-r_{p,1}\\ \ldots \\ r_{d,n}-r_{p,n} \end{array}\right]$$

Equation (2) is expressed as *a* · *β* = *b*. Using least squares, the algebraic solution of *β* is computed as *β* = (*a^T^* · *a*)^−1^ · *a^T^* · *b*. To improve the algebraic solution of *β* and the initial value of the distortion center *u*_0_, *v*_0_, the Levenberg-Marquart algorithm can be used. The Levenberg-Marquart algorithm solves non-linear least squares problems, especially in least squares curve fitting. A function to minimize and the initial values for the parameters set are necessary. In this case, the error function to be minimized is:

(8) $$J_{MP}=\frac{1}{n}\cdot {\sum_{i=1}^{n}\left(r_{p,i}-\frac{r_{d,i}}{1+\beta_{0}\cdot r_{d,i}^{2}+\beta_{1}\cdot r_{d,i}^{4}}\right)}^{2}$$

This error function measures the mean radial distance between computed undistorted control points and distorted control points corrected with a given model. The set of parameters are *β*_0_, *β*_1_ and the distortion center *u*_0_, *v*_0_. Initial values for *β*_0_, *β*_1_ are the solution of (7) and *u*_0_, *v*_0_ is the center of the image. The minimization of the error function (8) computes the best values for *β*_0_, *β*_1_, *u*_0_, *v*_0_.

If a first-order division model is computed with only one parameter *β*_0_, expression (7) is reduced as follows:

(9) $$\left[\begin{array}{c} r_{p,1}\cdot r_{d,1}^{2}\\ \ldots \\ r_{p,n}\cdot r_{d,n}^{2} \end{array}\right]\cdot \beta_{0}=\left[\begin{array}{c} r_{d,1}-r_{p,1}\\ \ldots \\ r_{d,n}-r_{p,n} \end{array}\right]$$

In this case, expression (8) is also reduced as:

(10) $$J_{MP}=\frac{1}{n}\cdot {\sum_{i=1}^{n}\left(r_{p,i}-\frac{r_{d,i}}{1+\beta_{0}\cdot r_{d,i}^{2}}\right)}^{2}$$

To summarize, the image high distortion can be easily modelled with a first-order or second-order division model. To calibrate the division model, a set of distorted and undistorted points are needed. Distorted points are detected in the image and undistorted points are computed doing a metric correction taking into account that some constraints of the calibration template remain unchanged under perspective projection in a high distorted image. The calibration procedure is as follows:

1. A set of images from different camera-template distances are captured.
2. Control points are detected in captured images to obtain *q_d,i_*.
3. Control points are corrected to obtain *q_p,i_* using the procedure described in Section 2.1.1.
4. Using points of all images, camera parameters are computed with the algorithm described in Section 2.1.2. This step is very important because data coming from all images are used together to compute one lens distortion model that represents all of them. According to Figure 1, lens distortion is depth dependent. In consequence, to compute a unique model that represents lens distortion in images captured with different camera-template distances is not the best practice. To be rigorous, the depth dependence of the lens distortion has to be represented in the model to describe the distortion accurately.

### 2.2. Depth-Dependent Division Model Calibration

**Figure 4:**
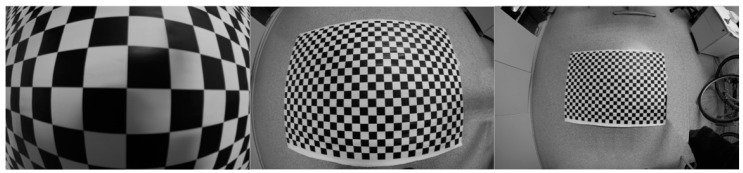
Checkerboard images taken at different camera-template distances. Checkerboard plane is as parallel as possible of the image plane. This guaranties that all control points are in the same camera-object distance and a unique valid distortion model represent them.

As it was shown in Figure 1, lens distortion is distance dependent. In consequence, to adjust a unique model using data of images taken at different distances to the camera is not a very good practice. Moreover, images with different perspective give control points at different distances to the camera that fail in validating the calibration result. If so, biased parameters are computed. To obtain a lens distortion model that represent the true image deformation, this should depend on the camera-object distance. This means that all lens distortion model parameters should be dependent on the camera-template distance. In this case, a first- and second-order division model is adjusted. Therefore, parameters *β*_0_, *β*_1_ have to depend on the camera-object distance. To solve this problem, two functions *β*_0_(*d*) and *β*_1_(*d*) are proposed that give the value of the parameter *β*_0_, *β*_1_ for a given camera-object distance. In this case, the depth-dependent second-order division model is as follows:

(11) $$r_{p}=\frac{r_{d}}{1+\beta_{0}(d)\cdot r_{d}^{2}+\beta_{1}(d)\cdot r_{d}^{4}}$$

where functions *β*_0_(*d*) and *β*_1_(*d*) represent the parameter *β*_0_, *β*_1_ for a given distance. To adjust the functions *β*_0_(*d*) and *β*_1_(*d*), an empirical experiment is proposed. Several checkerboard images are taken at different camera-template distances. Camera location in front of the checkerboard is adjusted to guarantee that the checkerboard plane is as parallel as possible to the image plane to avoid perspective that can influence in a bad result. This guaranties that all control points are in the same camera-object distance and a unique valid distortion model represents them. A set of images at different camera-checkerboard distances are taken to compute one parameter *β*_0_, *β*_1_ with every image. This is the main difference with the method to compute just one model to represent the lens distortion with independence to the camera-template distance. With one model, all control points from all images participate in the calibration of the model. Now, only control points coming from one image captured in a specific camera-template distance are used to compute one model that is valid for this distance. Using control points coming from images captured at different camera-template distances, a set of models are computed that are valid for a specific distance. The aim is to define functions *β*_0_(*d*) and *β*_1_(*d*) that are able to represent the evolution of parameters *β*_0_, *β*_1_ throw the different models that are valid for different distances. Figure 4 shows one example of images taken under these conditions. A set of parameters *β*_0_, *β*_1_ are computed with every image taken at different distances that are used to adjust the function *β*_0_(*d*) and *β*_1_(*d*) empirically.

## 3. Results

**Figure 5:**
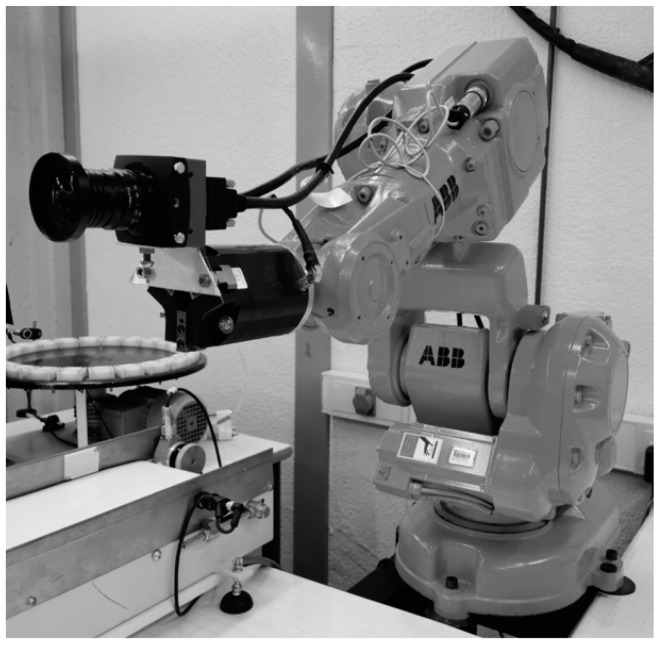
Robot arm ABB IRB 140 (ABB, Stuttgart, Germany) with an EoSens^®^ 12CXP+ camera of 4096 × 3072 pixels (Mikrotron, Unterschleißheim, Germany).

In our experiment, an EoSens^®^ 12CXP+ (Mikrotron, Unterschleißheim, Germany) of 4096 × 3072 pixels, 23.04 × 23.04 mm active area is assembled on an ABB IRB 140 showed in Figure 5. Camera location is obtained by using the location of the end of the robot arm that is provided by its control unit. With this location, it is possible to know the camera-template distance and moreover, it is possible to orientate the camera sensor plane with the template plane to be as much parallel as possible. Two lenses with 8 mm and 12 mm manual focus are mounted on the camera to compare the results. The training model plane is a 1210 mm × 970 mm checkerboard with 580 corner points (29 × 20). Corner detection has been done with the function cvFindCheckerboardCorners() from the openCV library [15]. Figure 3 shows distorted detected points in one image and their computed undistorted position.

Ten images, similar to the ones in Figure 5, are taken at several camera-checkerboard distances with both lenses. Distances are from 300 mm to 1650 mm in steps of 150 mm. Twenty division models are computed, one for each image and for each lens. The process is as follows:

1. The checkerboard control points are detected in each image to obtain *q_d,i_*.
2. Distorted control points *q_d,i_* are undistorted to obtain *q_p,i_* using the metric correction method described in Section 2.1.1.
3. Detected control points in each image *q_d,i_* and undistorted points in each image *q_p,__i_* are used to adjust a particular division model for each image. A division model is adjusted as follows:
  - The algebraic solution is computed solving Equation (7) and considering as the distortion center the center of the image.
  - Model parameters and distortion center are refined minimizing the error function defined in (8).

**Figure 6:**
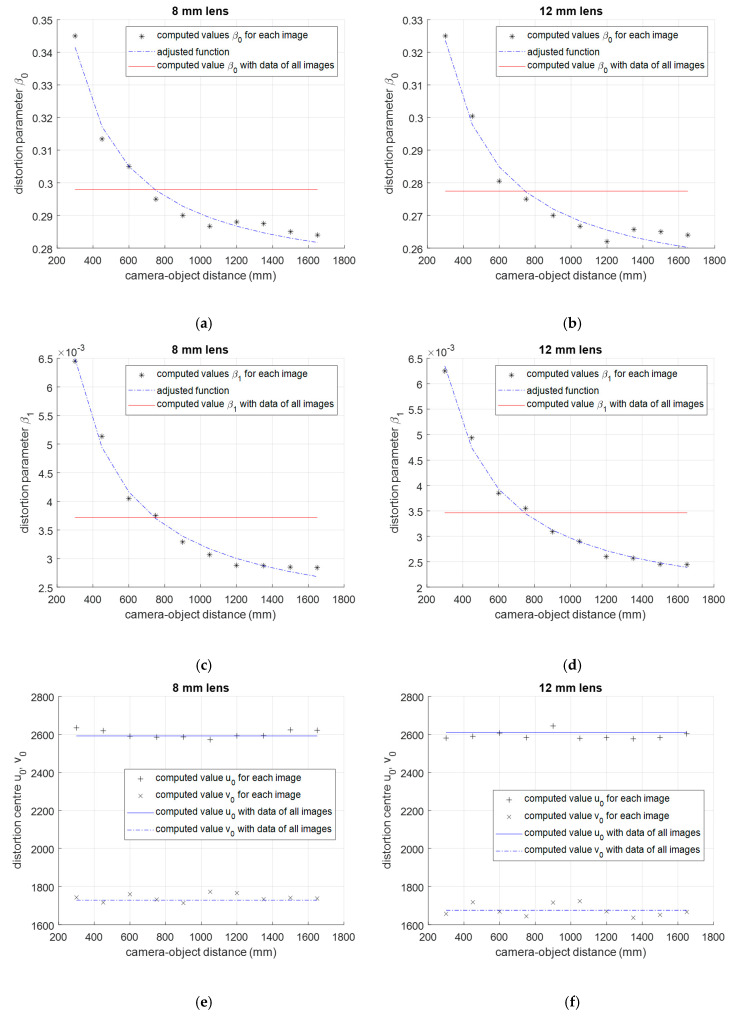
Variation of the second order lens distortion model parameters with the camera-object distance. The computed parameters are *β*_0_, *β*_1_ and distortion center *u*_0_, *v*_0_. The asterisks show the computed value of the parameter for each image and the dashed line shows the value given by the function *β*_0_(*d*), *β*_1_(*d*) adjusted using the least squares technique with asterisk data, *β_0i_* and *β_1i_*. (**a**,**c**,**e**) is for 8 mm lens and (**b**,**d**,**f**) is for 12 mm lens.

One particular division model represents the distortion for one camera-template distance. The method to compute the model is described in Section 2.1.2, but in this case, control points of one image are used instead of using control points of several images. The computed parameters with each model are *β*_0_, *β*_1_ and the distortion center *u*_0_, *v*_0_, that are valid for one distance. Figure 6 shows the computed parameters for each image for 8 mm and 12 mm lenses. The variation of lens distortion parameters depending on the distance is similar if 8 mm or 12 mm lens are used. Distortion center *u*_0_, *v*_0_ does not change significantly when the camera-checkerboard distance varies, but distortion parameters *β*_0_, *β*_1_ change as it is shown in Figure 6a–d. As it was presented by Magill in [3] and shown in Figure 1, distortion is depth-dependent and distortion parameters are sensitive to the camera-object distance. The asterisks in Figure 6a–d show the computed value of parameter *β*_0_, *β*_1_ for each image and both lenses respectively. According to the sensibility of parameters *β_0i_* and *β_1i_* to the distance, the values of the lens distortion parameters decreases with the distance in an exponential form. In consequence, the following experimental function can represent the variation of the distortion model parameter *β*_0_, *β*_1_ with the distance *d*:

(12) $$\beta (d)=\frac{k_{1}}{d}+k_{0}$$

*k*_0_ and *k*_1_ are the adjustable parameters. The aim is to represent the depth dependent variation of camera lens distortion parameters with the simplest function that is possible to describe this phenomenon. A mathematical analysis of this phenomenon would result in a more complex equation with more parameters to identify, but from a practical point of view, the empirical analysis shows that the two parameters equation proposed in (12) are enough to describe this phenomenon. The dashed lines in Figure 6a–d show the value given by the functions *β*_0_(*d*) and *β*_1_(*d*) adjusted with the least squares technique using data *β_0i_* and *β_1i_* represented with asterisks. In both cases, using 8 mm lens or 12 mm lens, the variation of distortion parameters with camera-object distance is represented accurately. A more complex function presented in (12) does not improve the results significantly and it would only complicate the computing process. The function proposed in (12) is able to describe this phenomenon accurately in both cases.

If control points of all images were used to compute the distortion parameter *β*, only one division distortion model would be computed for all images. Computed value is represented with a solid red line in the Figure 6a–d.

In addition, to simplify the lens distortion model also, a first-order division model is computed with the same data. In this case, the aim is to know how the division model degree can improve the distortion correction capacity of the model. The procedure is similar as before but in this case the division model is represented by the distortion center *u*_0_, *v*_0_, and one parameter *β*_0_ only, instead of two *β*_0_ and *β*_1_ as before. The first degree depth dependent division model is defined as follows:

(13) $$r_{p}=\frac{r_{d}}{1+\beta_{0}(d)\cdot r_{d}^{2}}$$

**Figure 7:**
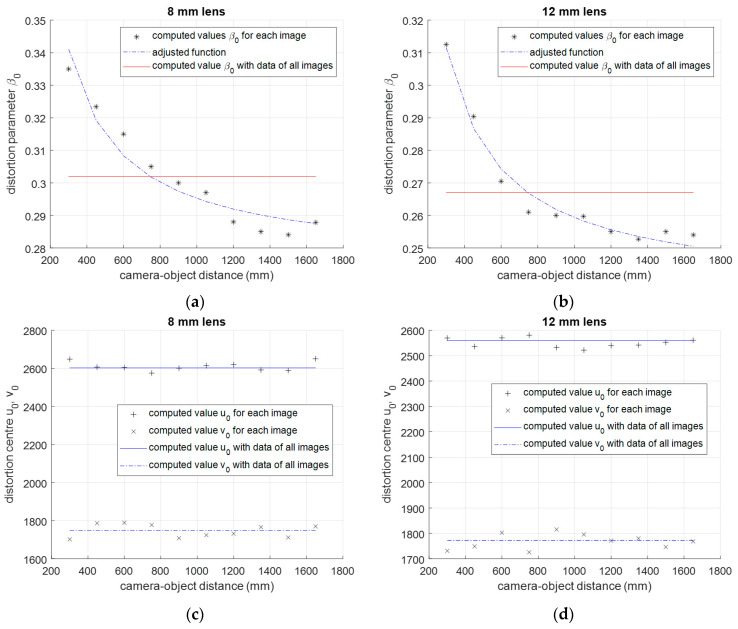
Variation of first-order lens distortion model parameters with the camera-object distance. (**a**,**b**) Computed parameter *β*_0_ for 8 mm and 12 mm lens respectively. The asterisks show the computed value of parameter *β*_0_ for each image and the dashed line shows the value given by the function *β*_0_ (**d**) adjusted using the least squares technique with asterisk data *β_0i_*. (**c**,**d**) Distortion center *u*_0_, *v*_0_ for 8 mm and 12 mm lens respectively.

Using corrected and distorted control points of each image, initial value of *β*_0_ is computed with Equation (9) and final values of *β*_0_ and *u*_0_, *v*_0_ are computed with Equation (10). Figure 7 shows that the variation of *β*_0_ with respect to the camera-object distance is similar as before. In consequence, Equation (12) is also used to represent the depth dependency of lens distortion parameters with the camera-object distance. Asterisks in Figure 7a,b show the computed value of parameter *β*_0_ with each image and both lenses. The blue dashed line in the Figure 7a,b is the computed values of function represented in (12) when it is adjusted using data *β_0i_* represented with asterisks. The solid red line in the Figure 7a,b represents the computed parameter *β*_0_ using control points of all images together.

To compare the accuracy of the proposed method the error function defined in (8) and (10) is used. Equation (8) is for the second-order division model and Equation (10) is for the first-order one. This function measures the mean error radial distance between computed undistorted control points and distorted control points detected in the image and corrected with a given model. Compared models are the unique model computed with data of all images, the first-order depth-dependent division model and the second-order depth-dependent division model. All of them are compared with 8 mm and 12 mm lens. Results are shown in Table 1 for 8 mm lens and Table 2 for 12 mm lens. First and second row show the computed error using the second and first-order depth dependent division model for each image respectively. Third row shows the error using a unique division model that undistorts all images with independence of the camera-object distance. Analyzing 8-mm lens results, the error has a mean value from 5.08 to 2.45 with a standard deviation from 2.92 to 1.07 pixels using the depth-dependent model depending on the model order. If a unique model is used it increases from 9.85 to 7.73 with and standard deviation from 4.83 to 2.98. The depth dependent computed model projects all undistorted points in a range of ±2.92 pixels in the worst case and the unique model does it in a range of ±4.83 pixels. Depth-dependent model represents the distortion accurately and a second-order model does not improve the image correction significantly. Similar results are for 12 mm lens.

The proposed method suggests that the camera sensor plane has to be as parallel as possible to the template plane in order to be sure that the camera-template distance is similar at all areas of the image. To analyze the plane parallelism effects in the calibration process, an experiment is performed that captures images where the camera sensor plane is not parallel to the template plane. Small errors are induced in the capturing stage. In this case a first order division model is computed with images captured with 8 mm lens. Calibration deficiencies are measured using Equation (10). Values are shown in Table 3. If they are compared with error values computed in Table 1 where both planes were parallel, no significant difference exists.

## 4. Discussion

Attending to the results presented in Table 1 and Table 2, the depth dependent distortion model is able to correct the image more accurately if it is compared to the non-depth-dependent model. The depth dependence is not considered if a unique model is computed using data from several images captured in different camera-object distances. In consequence, a biased model is computed that does not represent the distortion accurately taking into account that lens distortion is a depth-dependent phenomenon.

The variation of lens distortion parameters with the camera-object distance can be described with the equation proposed in (12) easily. A more complex function does not improve the results significantly. Going deeper, the Equation (12) is able to represent variation of lens distortion parameters when the model changes from first to second degree or when the lens changes. In this case, it has been validated for 8 mm and 12 mm lenses.

From the point of view of the degree of the division model, a first-order degree model corrects the image distortion acceptably but it can be improved with a second-order model if necessary.

With the proposed method, images are captured without perspective to have similar camera-checkerboard distance in all parts of the image. Since the image plane is as parallel as possible to the camera sensor plane, image focusing is easier and all control points in the image are focused. This improves the detection of control points in the image and reduces the noise level in the calibration process. With existing methods, the image perspective makes the focusing process harder and some parts in the image are blurred.

It may be considered that the proposed method is useless because it needs special equipment to perform the lens distortion calibration. In this case, a robot arm is used to localize the camera and to measure the camera-checkerboard distance. However, similar results are computed if the camera is localized with a tripod and camera-object distance is measured. As it is demonstrated in Table 3, perfect parallelism between the camera sensor plane and the checkerboard plane is not definitive to obtain accurate results.

## 5. Conclusions

A deep analysis of the high distortion image correction has been done. In this case, the analysis is focused in the distance dependence of the distortion in the image. The depth dependence of lens distortion model was presented by Magill in [4] and this paper presents an analysis in high distorted images. A variation of the camera lens distortion division model is presented that includes the camera-object distance to obtain accurate results as it is shown in Table 1, Table 2 and Table 3. The division model is able to correct high distorted images with only one parameter. This parameter can be adjusted to the camera-object distance to perform an accurate correction of high distorted images. Better results are computed if they are compared to the image correction, if only one model is computed that does not depend on the camera-object distance. The distortion center parameters do not vary significantly if the camera-object distance changes. At present, the proposed analysis is a step forward in the field of high lens distortion correction that will help in any application where the lens distortion correction represents a crucial step.

## Figures and Tables

**Table 1:** Calibration error with 8-mm lens and parallel planes (mean and standard deviation) *^a^*.

	Camera-Object Distance (mm)									
	300	450	600	750	900	1050	1200	1350	1500	1650
Second order depth-dependent model	4.75 ± 2.23	4.86 ± 2.38	4.25 ± 2.83	3.45 ± 1.85	3.04 ± 1.53	3.37 ± 1.63	3.15 ± 1.07	2.45 ± 1.32	2.99 ± 1.52	3.01 ± 1.58
First order depth-dependent model	4.34 ± 2.37	5.08 ± 2.88	4.03 ± 2.92	3.27 ± 2.08	3.63 ± 2.05	3.85 ± 1.83	2.99 ± 1.14	2.83 ± 1.06	2.73 ± 1.17	3.18 ± 1.03
Unique model	8.16 ± 4.73	7.29 ± 4.83	8.94 ± 4.23	9.85 ± 3.72	9.14 ± 3.36	8.63 ± 3.23	7.73 ± 3.32	8.94 ± 3.09	8.45 ± 2.98	7.79 ± 3.01

*^a^* Calibration error is the evaluation of the error function defined in Equation (8) for the second-order model and Equation (10) for the first-order model. It measures the mean of the radial distance between the undistorted points *q_p_* used to calibrate the model and the detected points *q_d_* undistorted with the calibrated model.

**Table 2:** Calibration error with 12-mm lens and parallel planes (mean and standard deviation) *^a^*.

	Camera-Object Distance (mm)									
	300	450	600	750	900	1050	1200	1350	1500	1650
Second order depth-dependent model	5.85 ± 3.32	4.63 ± 2.57	4.62 ± 2.62	3.38 ± 2.04	2.98 ± 2.13	3.05 ± 1.86	3.23 ± 1.57	2.56 ± 1.14	2.63 ± 1.38	2.79 ± 1.49
First order depth-dependent model	4.72 ± 3.01	4.98 ± 2.63	4.41 ± 2.98	3.43 ± 2.52	3.36 ± 2.82	3.58 ± 2.81	2.97 ± 1.74	2.43 ± 1.57	2.57 ± 1.05	2.63 ± 1.14
Unique model	8.96 ± 5.82	8.09 ± 5.94	8.16 ± 4.94	9.64 ± 4.82	9,52 ± 4.82	9.72 ± 3.95	8.62 ± 4.39	7.89 ± 3.92	9.94 ± 3.67	8.82 ± 3.73

*^a^* Calibration error is the evaluation of the error function defined in Equation (8) for the second-order model and Equation (10) for the first-order model. It measures the mean of the radial distance between the undistorted points *q_p_* used to calibrate the model and the detected points *q_d_* undistorted with the calibrated model.

**Table 3:** Calibration error with 8 mm lens and non-parallel planes (mean and standard deviation) *^a^*.

	Camera-Object Distance (mm)									
	300	450	600	750	900	1050	1200	1350	1500	1650
First order depth-dependent model	4.88 ± 2.83	4.75 ± 2.62	4.15 ± 2.39	3.55 ± 2.02	3.17 ± 2.12	3.27 ± 1.94	3.38 ± 1.35	2.91 ± 1.27	2.87 ± 1.48	3.27 ± 1.31
Unique model	9.34 ± 5.73	8.02 ± 4.63	9.04 ± 5.36	9.75 ± 4.97	9.37 ± 4.05	8.03 ± 3.95	8.16 ± 3.78	8.03 ± 3.65	9.82 ± 3.89	8.71 ± 4.31

*^a^* Calibration error is the evaluation of the error function defined in Equation (10). It measures the mean of the radial distance between the undistorted points *q_p_* used to calibrate the model and the detected points *q_d_* undistorted with the calibrated model.

## References

[1] Hartley R., Zisserman A. (2000). Multiple View Geometry in Computer Vision.

[2] Ricolfe-Viala C., Sanchez-Salmeron A. (2010). Lens distortion models evaluation. Appl. Opt..

[3] Wieneke B. (2008). Volume self-calibration for 3D particle image velocimetry. Exp. Fluids.

[4] Magill A.A. (1955). Variation in distortion with magnification. J. Opt. Soc. Am..

[5] Fryer J.G., Brown D.C. (1986). Lens distortion for close-range photogrammetry. Photogramm. Eng. Rem. S.

[6] Fryer J.G., Fraser C.S. (1986). On the calibration of underwater cameras. Photogramm. Rec..

[7] Fraser C.S., Shortis M.R. (1992). Variation of distortion within the photographic field. Photogramm. Eng. Remote Sens..

[8] Alvarez L., Gómez L., Sendra J.R. (2011). Accurate depth dependent lens distortion models: An application to planar view scenarios. J. Math. Imaging Vis..

[9] Ricolfe-Viala C., Sanchez-Salmeron A., Martinez-Berti E. (2012). Accurate calibration with highly distorted images. Appl. Opt..

[10] McGlone C., Mikhail E., Bethel J. (2004). Manual of Photogrammetry.

[11] Fitzgibbon A. Simultaneous linear estimation of multiple view geometry and lens distortion. Proceedings of the Conference on Computer Vision and Pattern Recognition, IEEE Computer Society.

[12] Claus D., Fitzgibbon A. A rational function lens distortion model for general cameras. Proceedings of the International Conference on Computer Vision and Pattern Recognition, IEEE Computer Society.

[13] Ricolfe-Viala C., Sanchez-Salmeron A.J. (2010). Robust metric calibration of non-linear camera lens distortion. Pattern Recogn..

[14] Devernay F., Faugeras O. (2001). Straight lines have to be straight. Mach. Vis. Apps..

[15] Bradski G., Kaehler A. (2008). Learning OpenCV.

